# Antimicrobial and Antibiofilm Activity against* Streptococcus mutans* of Individual and Mixtures of the Main Polyphenolic Compounds Found in Chilean Propolis

**DOI:** 10.1155/2019/7602343

**Published:** 2019-01-02

**Authors:** Jorge Jesús Veloz, Marysol Alvear, Luis A. Salazar

**Affiliations:** ^1^Departamento de Ciencias Biológicas y Químicas, Facultad de Medicina y Ciencia, Universidad San Sebastián, Campus Los Leones, Lota 2465, 7510157 Providencia, Santiago, Chile; ^2^Departamento de Ciencias Químicas y Recursos Naturales, Facultad de Ingeniería y Ciencias, Universidad de La Frontera, Avenida Francisco Salazar 01145, 4811230 Temuco, Chile; ^3^Center of Molecular Biology and Pharmacogenetics, Scientific and Technological Bioresource Nucleus (BIOREN), Universidad de La Frontera, Avenida Francisco Salazar 01145, 4811230 Temuco, Chile

## Abstract

Dental caries is multifactorial disease and an important health problem worldwide.* Streptococcus mutans* is considered as a major cariogenic agent in oral cavity. This bacteria can synthetize soluble and insoluble glucans from sucrose by glucosyltransferases enzymes and generate stable biofilm on the tooth surface. Biological properties of Chilean propolis have been described and it includes antimicrobial, antifungal, and antibiofilm activities. The main goal of this study was to quantify the concentrations of main flavonoids presents in Chilean propolis and compare some biological properties such as antimicrobial and antibiofilm activity of individual compounds and the mixture of this compounds, against* S. mutans *cultures. Chilean propolis was studied and some polyphenols present in this extract were quantified by HPLC-DAD using commercial standards of apigenin, quercetin, pinocembrin, and caffeic acid phenethyl ester (CAPE). MIC for antimicrobial activity was determined by serial dilution method and biofilm thickness on* S. mutans *was quantified by confocal microscopy. Pinocembrin, apigenin, quercetin, and caffeic acid phenethyl ester (CAPE) are the most abundant compounds in Chilean propolis. These polyphenols have strong antimicrobial and antibiofilm potential at low concentrations. However, pinocembrin and apigenin have a greater contribution to this action. The effect of polyphenols on* S. mutans* is produced by a combination of mechanisms to decrease bacterial growth and affect biofilm proliferation due to changes in their architecture.

## 1. Introduction

Dental caries is an infectious disease that affects people from developed and underdeveloped countries. In Chile, it is an important health problem that affects adults and children from a low socioeconomic status [1]. This multifactorial disease leads to tooth destruction and removing the enamel by means of degradation of mineral material.

*Streptococcus mutans* (*S*.* mutans*) has been indicated as the major cariogenic agent in oral cavity. Such bacteria can synthetize soluble and insoluble glucans from diet sucrose mediated by glucosyltransferases enzymes (gtfs), which allows extracellular aggregation for stable biofilm formation on the tooth surface [2–4]. However, gtfs are encoded by GTFB, GTFC, and GTFD genes and previous studies have demonstrated that polyphenols-rich extract of Chilean propolis decreases GTFs gene expression levels and exerts a functional effect on its enzymatic capacity for synthetizing insoluble glucans at subinhibitory concentrations [5].

The biological properties of propolis have been established from some years and include antifungal, antiatherogenic, antioxidant, and antimicrobial activities [6–8]. High content of polyphenols in Chilean propolis can inhibit the growth of the* S. mutans* and reduce biofilm formation without bactericidal effect [9, 10]. Moreover, polyphenols from Chilean propolis can affect the expression of genes involves in* S*.* mutans* virulence and the capacity for forming a biofilm [11, 12].

The composition of Chilean propolis extract from La Araucanía Region showed high concentrations of polyphenols and the presence of different families of flavonoids with pinocembrin being the predominant compound [13].

This study aimed to compare the antimicrobial activity of the individual polyphenols quantified in Chilean propolis and a mixture of this compounds applied to* S. mutans* cultures. Thus, we evaluated different concentrations in a mixture of these compounds to confirm its synergistic action in order to describe this effect as a main property of polyphenols to change a biofilm structure and affect its size as additional factor to decrease the virulence of this microorganism.

## 2. Materials and Methods

### 2.1. Preparation of Polyphenol-Rich Extract of Propolis (EP)

To evaluate the effect of polyphenols from EP in* S. mutans* antimicrobial activity and biofilm formation, the propolis was collected during the spring of 2008 from La Araucanía Region (Chile). Propolis crude sample was kept frozen (-20°C) and later crushed in cold, and 30 grams was dissolved in 100 mL of ethanol (70%) and macerated for 7 days at room temperature. The ethanolic extract of propolis (EEP) was filtered with Whatman 2.0 paper and centrifuged at 327 g, during 20 minutes at 5°C. Finally, the solvent was evaporated at a temperature of 40°C, for 2 hours in a Rotavaporator (Buchi, R-210, Germany) and dissolved for 24 h with sterile DMSO (0.01%) to obtain Polyphenol-rich Extract of Propolis (EP).

### 2.2. Determination of Total Phenolic Content in EP

The content of total polyphenols in EP was quantified by Folin-Ciocalteau reaction by a modification of Popova and collaborator's methodology [14]. For this assay, 100 *μ*L of EP was mixed with 100 *μ*L of distilled water and 2 mL of Folin-Ciocalteau reagent (Merck, Germany). The resulting solution was incubated for 8 minutes, and finally 3 mL of sodium carbonate 20% (w/v) was added. The absorbance of this solution was measured at 760 nm after 2 hours of incubation at room temperature. The concentration of polyphenols was calculated from a calibration curve and was expressed in mg mL^−1^ equivalent to the pinocembrin-galangin standard mixture 1:1.

### 2.3. Identification and Quantification of Polyphenolic Compounds Present in EP

Four compounds were identified and their concentrations were calculated by the direct injection method in a liquid chromatograph of high resolution (Shimadzu, Japan), equipped with a LC-20AT pump connected to a UV-Visible detector SPD-M20A UV (HPLC-DAD). The separation was carried out in a LiChrospher RP-18 column, with particle size 5 *µ*m x 250 mm and stove CTO-20AC at 25°C. The elution was realized at 40°C using a acetonitrile, methanol, water, and formic acid 5% mixture in a flow of 1.0 ml min^−1^, with a gradient from 30 to 70% and 20 *µ*L of the diluted EP sample (1:50) were injected. To calculate compounds concentrations, we used solutions at 5 ppm of apigenin, quercetin, pinocembrin, and caffeic acid phenethyl ester (CAPE) under commercial standards (Sigma-Aldrich, St Louis, MO), and the concentrations were expressed in mgL^−1^ by interpolation in a calibration curve (see Table 1).

### 2.4. Cellular Culture Conditions and Biofilm Generation

S*. mutans* strains were obtained from samples of fluids of the oral cavity from children with tooth decay. The cultures were made in Petri plates. Biofilm samples for studies were obtained after inoculation of 5 x 10^5^ UFC mL^−1^ in 96-well microplates. For confocal microscopy studies, the biofilm was obtained in Fluorodish microplates (World Precision Instrument Inc., China). All cultures were incubated for 24 hours, at 37°C and 5% of CO_2_ with Trypticase Soy (TSB) (Becton Dickinson and Co, NY, USA) and sucrose (1%) in a container (Anaerobic Generator GasPak EZ (Becton, Dickinson and Co., NY, USA). The presence of* S. mutans* was confirmed by polymerase chain reaction (PCR) as previously described [15].

### 2.5. Antimicrobial Activity of Individual and Mixtures of Polyphenols

The Minimum Inhibitory Concentration (MIC) was determined by serial dilution method following the CLSI guidelines [16]. The strains of* S. mutans* suspension 5 x 10^5^ CFU mL^−1^ were inoculated in a 96–well microplates containing 100 *μ*L of TSB and sucrose 1% supplied with 100 *μ*g mL^−1^ of EP or 25 *μ*g mL^−1^ of commercial polyphenols in DMSO (0.1%), respectively. We used different controls: a positive control 10 *μ*L of chlorhexidine digluconate (0.2%) and a control without propolis, as a negative control (vehicle) was included. All tests were run in triplicate.

### 2.6. Evaluation of Biofilm Formation

Biofilm samples were prepared in FluoroDish microplates in TSB and sucrose 1%, polyphenols at 25 *µ*g mL^−1^, and a mix of polyphenols (apigenin, pinocembrin, quercetin, and CAPE), in concentrations of 6.25; 12.5 and 25 *µ*g mL^−1^. The bacterial biofilm was incubated for 1 hour at room temperature with 100 *µ*L of the probe Calcein Biofilm Tracer™ (Invitrogen, the USA), and later it was washed with sterile PBS. The structure of the biofilm was observed by a confocal microscope Olympus Fluoview 100, equipped with lens of watery immersion (To x 60, 0.21 NA). The images were captured by means of direct acquisition by excitement at 480 nm and the sign of fluorescence was detected by means of the green channel. The confocal planes for the three-dimensional image obtained in intervals of 15 seconds and 0.5 *μ*m for plane and a scan of 512 x 512 pixels. The images were processed in the software ImageJ Mac Biophotonic.

### 2.7. Statistical Analysis

Statistical analyses were performed using the computational software package Prism 5 (Graph Pad Software Inc., San Diego, USA). Experimental values of MIC means obtained from antimicrobial test were estimated by statistical analysis of variance (ANOVA) and a Posttest of Tukey. To compare the values from individual's experiments of biofilm size, we applied unpaired two-sample* t*-tests statistical analysis.* P* < 0.05 was considered statistically significant.

## 3. Results

### 3.1. Total Polyphenols Content in Chilean Propolis and Compounds Quantification

In EP the entire polyphenols contents in equivalence of pinocembrin-galangin mixture were quantified by Folin-Ciocalteau reaction and it was 137.7 ± 0.7 mg g^−1^. This result is concordant with similar results from other studies [14, 17].

**Figure 1:**
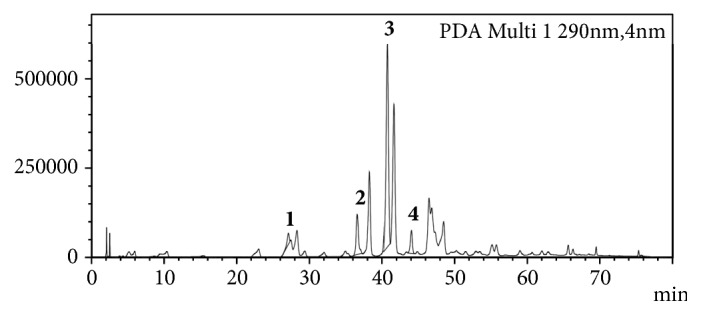
Compounds identified in EP2008 by HPLC-DAD. 1. Quercitin, 2. Apigenin, 3. Pinocembrin, and 4. CAPE.

The main flavonoids were identified on the Chilean propolis by means of the HPLC-DAD as shown in Figure 1. In accordance with the times of retention (Tr) and the direct comparison with commercial standards, it was possible to verify the presence of quercetin, apigenin, pinocembrin, and caffeic acid phenethyl ester (CAPE). Concentrations of these polyphenols were quantified by HPLC-DAD (Table 2).

### 3.2. Antimicrobial Activity of Individual and Mixtures of Polyphenols Found in Propolis

An antimicrobial test was carried out in order to quantify MIC in bacteria cultures submitted to treatment with solutions of some polyphenols identified in the EP at the same concentrations (25 *µ*g mL^−1^). The values of MIC for* S. mutans* when the plates supplied with Polyphenols Mixture exhibit a similar potential as traditional chlorhexidine (1.6 *μ*g mL^−1^).  Table 3 shows that flavonoids, as apigenin and pinocembrin, had lower values of MIC when compared with a mixture. All treatments showed significant statistical differences in comparison of quercetin and CAPE (p < 0.5 and p < 0.001, respectively).

### 3.3. Effect of the Polyphenols in the Reduction of Bacterial Biofilm Thickness

**Figure 2:**
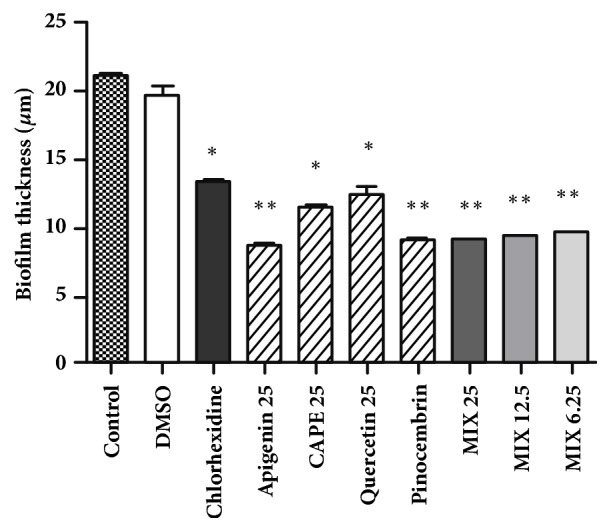
Evaluation of bacterial biofilm thickness in* S. mutans* cultures treated with individual and mixtures of polyphenols. Each values of individual experiments were expressed as a mean ± standard deviation. P-value was determined by ANOVA and Tukey Post Test. *∗*p < 0.5; *∗∗*p<0.01, when compared with the control without treatment.

**Figure 3:**
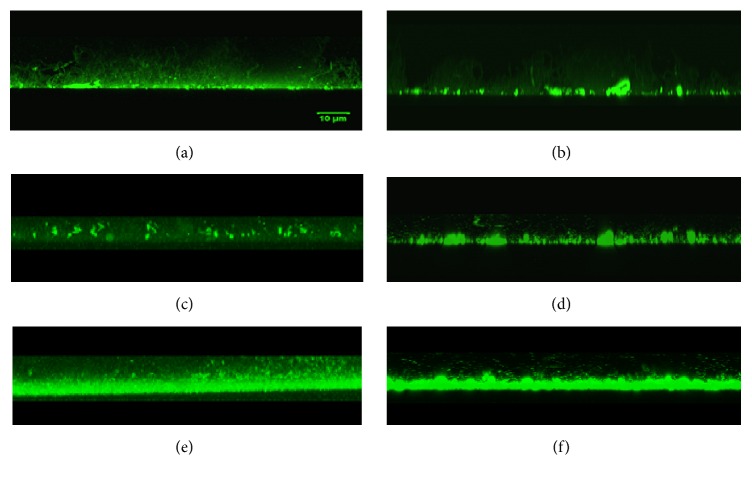
Images of bacterial biofilms from* S. mutans* cultures obtained by confocal microscopy. (a) Control group. (b) Chlorhexidine. (c) Pinocembrin. (d) Apigenin. (e) CAPE. (f) Quercitin.

Figure 2 represents results in the reduction on biofilm's architecture generated from cultures of* S. mutans*. As shown in Figure 3, the cariogenic bacteria produced a thinner biofilm in plates supplied with individual compounds and polyphenols mixtures. The biofilm generated by cells in the control group without antimicrobial treatment reached values higher than 20 *μ*m, as observed in confocal microscopy image of Figure 3(a). Samples treated with polyphenols mixture at low concentration (6.25 *μ*g mL^−1^) showed significant statistical differences (p< 0.01) when compared with untreated biofilm control. Again, some individual compounds in the solution, as pinocembrin (25 *μ*g mL^−1^) and apigenin (25 *μ*g mL^−1^) applied on* S. mutans *cultures, showed better potential in the reduction of the biofilm in comparison with a traditional synthetics products used for dental caries treatment as chlorhexidine (Figure 3(b)). The aspect and thickness of the biofilm that result from the bacterial cellular aggregation are detailed in Figures 3(c)–3(f).

## 4. Discussion

Since many years ago, the* S. mutans* has been identified as the main microorganism responsible for initiating the colonization of the oral cavity, and it is capable to generate an acidic ambience favorable for the degradation of tooth mineral and protein material [18]. The diversity in its biological activity has been investigated in numerous studies with high concentrations of entire polyphenols present in EP, especially certain types of polyphenols as flavonoids.

Previously, a seasonal effect has been demonstrated (collection time) on the Chilean propolis composition with changes in polyphenols families, identified in the samples where they were observed. Pinocembrin and apigenin were quantified in the EP extract obtained from La Araucanía. These polyphenols have been related to antimicrobial activities against* S. mutans* [19, 20].

The employment of chemical compounds as chlorhexidine in the treatment of oral diseases had not had entirely successful outcomes due to several local side effects as the bacterial tolerance, changes in teeth coloration, taste disorders, and alterations in oral cavity microorganism that provokes a resistance to the treatments in patients [21, 22]. These aforementioned circumstances make it necessary to conduct searches toward new substances with anticariogenic activity with higher antimicrobial potential and at the same time they do not cause toxicity for the human organism.

Certainly, the antimicrobial actions demonstrated in this study for some flavonoids as apigenin and pinocembrin have allowed us to obtain MIC values similar to the administration of chlorhexidine. Nevertheless, when only quercetin or CAPE was added, the values of MIC were very high (4.1 and 5.2 *μ*g mL^−1^, respectively). However, when four compounds were applied to the antibacterial treatments they then increased its antimicrobial polyphenol's effect. Although quercetin and CAPE were present in the mixture, the additive actions of polyphenols confirm these results, and also they can act at low concentrations. Prior to this study it was not possible to define which group of compounds had a greater contribution to this action.

Some studies showed that adherence of* S. mutans* in tooth surface depended on hydrophobicity and therefore affect cell-surface proteins as Antigen I/II involved in initials steps for adherence and biofilm formation [23]. In some of these investigations, the antimicrobial activity of the apigenin found in EP has been established and operates on the gtf C enzyme, and they prevent the synthesis of insoluble glucans for the formation of the extracellular matrix [24]. Actually the potential of that compound for disruption in biofilm accumulation and reduction of gtf D activity can explain its action in mind and late-exponential phase of bacteria growth [25, 26], but the mechanism of action of these polyphenolics compounds is not completely known yet.

Quercetin and its derivatives, isorhamnetin and quercetin-3-glucuronide, may reduce the expression of some inflammatory genes. Its effects have been described in cellular cultures on the oxigenase-1 protein and the transduction of nuclear factor NFkB also and the decrease in the expression of the gene Nrfk2 and the inactivation of miR-155 with proinflammatory activity [27]. In the case of the phenylates flavonoids identified in natives propolis of the Pacific Ocean, the strong antimicrobial potential is due to their direct effects on the bacterial cell membrane [28]. Quercetin and kaempferol, besides, have been related to inhibition of a glycolytic enzyme F-ATPase and subsequently increased the intracellular pH in* S. mutans* [29, 30].

One of the main virulence factors is the capacity for generating extracellular polymers (glucans) by means of glucosyltransferases activity in* S. mutans*. In this study, we also evaluate the potential of the polyphenols as agents that affect the formation of a stable bacterial biofilm. The growth process of the biofilm needs some steps that involve the cellular adherence to solid surfaces and the interactions of the cell-to-cell in microcolonies structures [31, 32].

The antimicrobials also produce changes in the structures of the biofilm and in the form of cellular aggregation due to changes in the levels of protein expression and enzymatic actions, but some phenolic acids, for example, caffeic acid and its phenethyl ester (CAPE), have a significant role in cancer cells apoptosis and cellular cycle, and it might affect bacterial multiplication [33, 34].

Confocal planes allowed visualizing changes in the architecture of the bacterial biofilm. A clear organization of the* S. mutans* biofilm architecture that grows upwards can be observed in the image of the control group, without treatment (Figure 3(a)). Flavonoids effects, such as pinocembrin and apigenin, are modifying the structures in the biofilm architecture of* S. mutans* (Figures 3(c) and 3(d), resp.). Figure 3(b) shows the reduction in thickness of the biofilm, while Figure 2 shows the significant statistical differences between polyphenols mixtures with respect to the control without treatment. Previous results demonstrate the additive action of polyphenols at small doses, achieving an inhibitory effect on biofilm formation [35]. The effect of pinocembrin and apigenin in relation to the control also showed a reduction in the biofilm thickness associated with enzymatic gtf C inhibition. Both compounds also have the highest antimicrobial activity, which are the flavonoids that contribute most to this action. So these mechanisms are very important for preventing tooth decay and consequently decrease* S. mutans* virulence and limiting extra oral colonization [36, 37]

These results demonstrate that the effect of polyphenols found in the Chilean propolis on* S. mutans* is produced by a combination of mechanisms, not only because their antimicrobial potential, since a significant reduction of the cellular adhesion and biofilm structure is achieved as an additional mechanism for tooth colonization.

## 5. Conclusion

These results suggest that polyphenols found in Chilean propolis exhibit antimicrobial activity against* S. mutans* at low concentrations. In addition, they decrease biofilm proliferation due to changes in their architecture. Moreover, pinocembrin and apigenin have strong antimicrobial and antibiofilm activity. This allows us to explain the contribution of these flavonoids to antimicrobial activity of polyphenols.

## Figures and Tables

**Table 1:** 

**Standard**	**LOD**	**LOQ**	**LR**	**Regression equation**	**R** ^**2**^
Quercetin	3.91	13.03	13.03 – 40.00	y = 16902x + 145755	0.9931
Apigenin	2.23	7.43	7.43 – 50.00	y = 59358x + 24261	0.9987
Pinocembrin	1.34	4.45	4.45 – 100.00	y = 151122x + 80657	0.9999
CAPE	0.29	0.98	0.98 – 20.00	y = 72216x - 84427	0.9998

LOD: limit of detection; LOQ: limit of quantification; LR: linear range; R^2^: coefficient of correlation

**Table 2:** Polyphenolics compounds quantified by HPLC-DAD in EP.

Compounds	Tr.	Area	Concentrations ± SD
	(minutes)	(mm^2^)	(mg L^−1^)
Apigenin	36.59	2409968	40.2 ± 0.6
CAPE	43.96	1234518	19.2 ± 0.3
Pinocembrin	40.66	12714319	83.6 ± 0.9
Quercetin	27.13	657840	21.0 ± 0.3

**Table 3:** Antimicrobial activity of individual compounds and polyphenols mixture on *S. mutans* cultures.

Compounds	MIC	p-value *vs* Quercetin	p-value *vs* CAPE
Polyphenols Mixture	1.6 ± 0.4	p<0.5	p<0.001
Apigenin	1.3 ± 0.4	p<0.01	p<0.001
Pinocembrin	1.4 ± 0.4	p<0.01	p<0.001
Chlorhexidine	1.6 ± 0.2	p<0.5	p<0.001
Quercetin	4.1 ± 0.8	-	NSD
CAPE	5.2 ± 0.8	NSD	-

MIC: minimum inhibitory Concentration. MIC values were expressed in *μ*g mL^−1^ as Mean ± Standard Deviation. P-value was calculated as significant differences after ANOVA Multiple Comparison and test Tukey's Posttest. NSD: Nonstatistical Differences.

## Data Availability

The data used to support the findings of this study are available from the corresponding author upon request.
